# Supplementary material for: Analyzing the Difference in the Length of Stay (LOS) in Moderate to Severe COVID-19 Patients Receiving Hydroxychloroquine or Favipiravir
Source: Pharmaceuticals (Basel). 2022 Nov 24;15(12):1456. doi: 10.3390/ph15121456 (PMC9785070; doi:10.3390/ph15121456)

Supplementary material online

Content

- Figure S1. Mirror plot for the propensity scores distribution balance between the two groups (both prior and after matching) after multiple imputation. 0=hydroxychloroquine and 1=Favipravir.

- Figure S2. Love's plot for the covariate balance between the two groups (prior and after matching) after multiple imputation.

Figure S1. Mirror plot for the propensity scores distribution balance between the two groups (both prior and after matching) after multiple imputation. 0=hydroxychloroquine and 1=Favipravir.

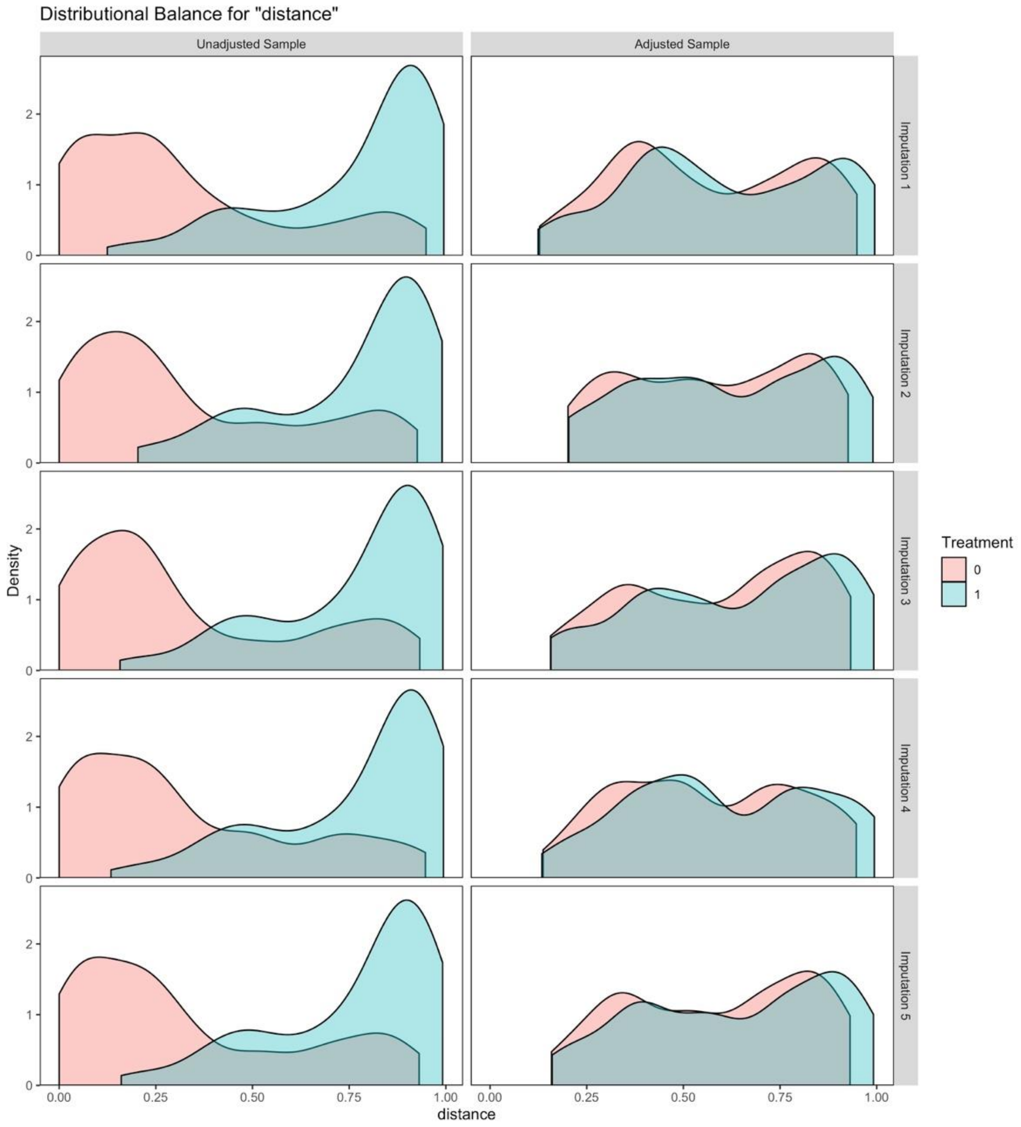

Figure S2. Love's plot for the covariate balance between the two groups (prior and after matching) after multiple imputation.

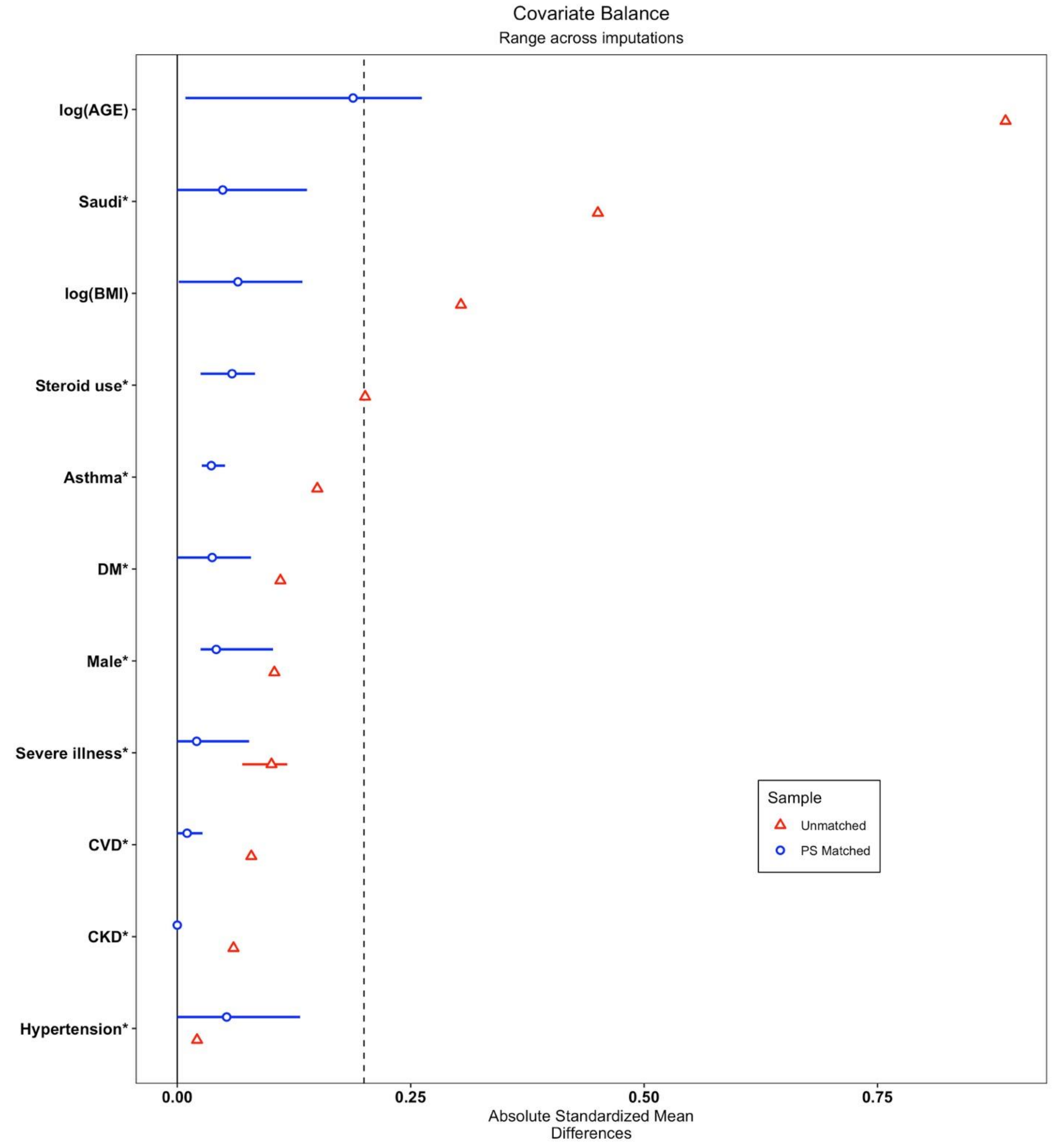

Supplement: Supplementary file 1 [file pharmaceuticals-15-01456-s001.zip › pharmaceuticals-2000758-supplementary.pdf]
